# Advances in circular RNAs and their roles in breast Cancer

**DOI:** 10.1186/s13046-018-0870-8

**Published:** 2018-08-29

**Authors:** Xuehui Wang, Lin Fang

**Affiliations:** 1 Nanjing Medical University, Nanjing, 211166 China; 2 Department of Thyroid and Breast Surgery, Shanghai Tenth People′s Hospital, Shanghai, 200070 China

**Keywords:** Circular RNA, Breast cancer, miRNA, Biomarker, Treatment

## Abstract

Circular RNAs (circRNAs) are a type of noncoding RNAs with a closed loop structure. With the development of high-throughput sequencing, massive circRNAs have been discovered in tumorous tissues. Emerging evidence suggests that the biological functions of circRNAs including serving as ceRNAs or miRNA sponges, interacting with proteins, regulating gene transcription and translation, suggesting that circRNAs will be novel biomarkers and targets for the diagnosis and prognosis of diseases. Breast cancer is the most frequently occurring cancer and the leading cause of cancer-related death among women worldwide. It is vital to understand the molecular pathways involved in the pathogenesis of proliferation and progression. In this review, we summarize the current knowledge on human circRNAs and their potential clinical implications on breast cancer.

## Background

Breast cancer is the most frequently occurring cancer and the leading cause of cancer-related death among women worldwide. It is estimated that there will be 255,180 new cases and 41,070 deaths of breast cancer in the United States in 2017 [1]. The last few decades have witnessed outstanding advances in breast cancer treatment and a large number of trials have been performed to find an effective treatment strategy, but the morbidity and mortality are still high. It has been proven that obesity [2], estrogen and progestin use [3], advanced maternal age at first birth [4] and alcohol consumption [5] are associated with an increased risk of breast cancer by Epidemiological studies. Besides factors above, genetic mutations and epigenetic mechanisms are also important in the tumorigenesis of breast cancer [6, 7]. Thus, it is vital to understand the molecular pathways involved in the pathogenesis of proliferation and progression. Over the last few years, reports have indicated that many ncRNAs, such as miRNAs, are involved in the carcinogenic process and can be used as biomarkers for early risk stratification and long-term survival prediction [8, 9]. As anthor novel class of endogenous ncRNA [10], the involvement of circRNAs in breast cancer has also been explored.

Circular RNA (circRNA) is a type of recently re-recognized RNA, the detected amounts and types of which are increasing at a rapid rate. CircRNA was first discovered in RNA viruses via electron microscopy in 1976 [11]. Subsequently, circRNAs were clearly observed in the eukaryotes by electron microscopy in 1979 [12]. They range in length from a few hundred to thousands of nucleotides [13]. Unlike linear RNAs that are terminated with 5’caps and 3’tails, circRNAs are single-stranded covalently closed circular transcripts [14]. Unfortunately, these circRNAs were thought to be the results of mis-splicing or by-products of pre-mRNA processing with low abundance and only a small number of circRNAs in different organisms have been identified over the past 30 years [15]. However, with the development and widespread use of high-throughput RNAsequencing (RNA-seq) technologies, as well as the development of specific algorithms for circRNA detection and quantification, circRNAs have recently been thrust into the spotlight as a newly appreciated class of non-coding RNAs. A large number of circRNAs have been successfully identified. Increasing evidence suggest that circRNAs are involved in the pathogenesis of a variety of diseases, such as neurological dystrophy [16], cardiovascular diseases [17] and cancer [18, 19]. In particular, circRNAs are reported to play important roles in tumorigenesis, metastasis, and therapy resistance [19]. Moreover, due to the stability and tissue specificity of circRNAs in blood or plasma [20–22], they could act as dependable diagnostic molecular biomarkers of hepatocellular carcinoma [23], gastric cancer [24] and son on.

In this review, we summarize the current knowledge on human circRNAs and present an overview of the potential clinical implications of human circRNAs on breast cancer.

## Circular RNAs

### Biogenesis and regulation of circRNAs

**Fig. 1:**
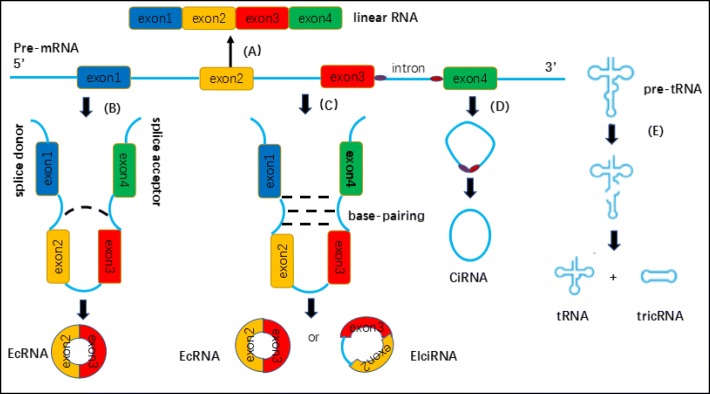
Biogenesis of circRNAs: (A) conventional splicing; (B) lariat-driven circularization; (C) intron-pairing-driven circularization; (D) intron cyclization; (E) the formation of tricRNA

Based on the components, circRNAs are mainly divided into three groups: exonic circRNAs (EcRNA) [25, 26], circular intronic RNAs (ciRNA) [27] and exon-intron circRNA (ElciRNAs) [28]. Today, the term ‘circRNA’ is commonly applied to describe exonic circRNAs consisting of one or more exons and predominantly remain in the cytoplasm [29]. EcRNA are synthesized by non-sequential backsplicing in which a downstream splice donor of pre-mRNA covalently joins with an upstream splice acceptor [30]. Two models for the origination of circRNAs by Jeck et al. are widely recognized, namely lariat-driven circularization (Fig. 1B) and intron-pairing-driven (Fig. 1C) circularization [31]. The formation of two models are similar except the first step. The former requires the covalent binding of the (5′) splice donor with the (3′) splice acceptor in the exon, while the latter involves complementary base-pairing across different introns especially between repetitive sequences such as ALU repeats to form a circular structure. Then these intronic sequences are subsequently trimmed to generate an ecRNA. Zhang et al. discovered a new type of circRNA in human cells that is derived from introns was termed circular intronic RNAs (ciRNAs), mainly found in the nucleus. The processing of such circular intronic RNAs (ciRNAs) depends on a consensus motif containing a 7 nt GU-rich element near the 5′ splice site and an 11 nt C-rich element close to the branchpoint site, resulting in a failure in debranching [27] (Fig. 1D). Very recently, Li et al. also found that in some circumstances, exons are circularized with introns’retained’ between exons. These circRNAs are termed exon-intron circRNAs or EIciRNAs [28]. EIciRNAs predominantly localize in the nucleus, interact with U1 snRNP and promote transcription of their parental genes. However, the mechanism of eIciRNA formation remains unknown.

Besides the circRNAs above, tRNA intronic circular RNAs (tricRNAs) are a class of abundant circular noncoding RNAs that are produced during metazoan tRNA splicing (Fig. 1E). This splicing mechanism is completely independent from that of pre-mRNAs. Biogenesis of tricRNAs requires anciently conserved tRNA sequence motifs and processing enzymes, and their expression is regulated in an age-dependent and tissue-specific manner [32, 33].

The biogenesis of circRNAs is regulated by various factors. Ashwal-Fluss et al. [32] have proved that circRNA are generated cotranscriptionally and their production rate is mediated by flanking intronic sequences [34]. Zhang et al. [26] demonstrated that exon circularization is dependent on flanking intronic complementary sequences. What’s more, exon circularization efficiency can be regulated by competition between RNA pairing across flanking introns or within individual introns. Adenosine deaminase 1 actingon RNA (ADAR1), a RNA-editing enzyme, could suppress circRNA expression by melting the stem structure [35]. That is, knockdown of ADAR1 induced elevated circRNA expression. A variety of RNA binding proteins (RBPs) have also been shown to induce the biogenesis of circRNAs. For instance, the alternative-splicing factor Quaking (QKI) protein was shown to modulate circRNA formation during the human epithelial–mesenchymal transition (EMT) [36]. Mouseblind (MBL/MBNL1), a splicing factor, is thought to promote its own circRNA biogenesis from the second exon in Drosophila and humans by bingding MBL-binding sites in flanking introns [34].

In brief, the biogenesis of circRNAs is still unclear and how these factors control the circRNA circulation remains to be further investigated.

### Functions of circular RNAs

Though the general mechanisms of circRNAs remain elusive, increasing evidences have reported that circular RNA participate in series of pathophysiology process.

#### CircRNAs serve as ceRNAs or miRNA sponges

**Fig. 2:**
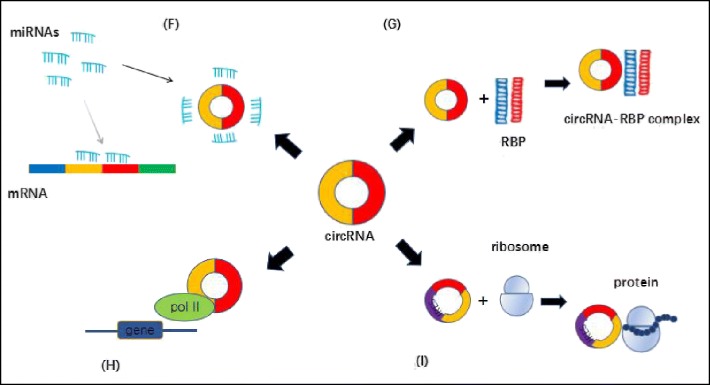
Functions of circRNAs: (F) miRNA sponges; (G) bind and sequester proteins; (H) regulate gene transcription; (I) translation

MicroRNAs (miRNAs) are important post-transcriptional regulators of gene expression that negatively regulate gene expression of messenger RNAs (mRNAs) through direct base pairing to target sites in mRNA 3’UTRs that leads to deadenylation, decreased mRNA stability and translation suppression [37]. The ceRNA hypothesis showed that other RNAs with miRNA target sites can compete with mRNAs for miRNA binding [38]. CircRNAs, containing a large number of different types of miRNA response elements MER (miRNA reponse element), have been found to interact with miRNA and serve as miRNA sponges in the cell, removing the inhibitory effect of miRNA on its target genes and further up-regulates the expression of the target genes [39] (Fig. 2F).

A circRNA named CDR1as, well known as ciRS-7, harboring more than 70 conserved binding sites and is highly expressed in human and mouse brains was first reported to function as a sponge of miR-7 [10]. Another circRNA called Sry (sex-determining region Y) was reported to serve as a sponge for miR-138 [39], thus regulating the invasion, development and metastasis of tumor cells. What’s more, a recently discovered circRNA, called cirITCH, similarly acts as a miRNA sponge via miR-7 and miR-20 [40]. All these findings above indicate that circRNAs could function as miRNA sponges to contribute to the regulation of cancers.

However, a recent study by Militello G et al. proved that few circRNA could function as ‘miRNA sponge’ [41]. Thus, as a classical model of circRNA function, ‘miRNA sponge’ is becoming more and more controversial.

#### CircRNAs bind and sequester proteins

CircRNA molecules could specifically bind to protein molecules directly or through RNA as well as sequester proteins to block the protein effects by working as competing elements (Fig. 2G). An example of the function of circRNA to interact with proteins is circFoxo3. CircFoxo3, preferentially expressed in the cytoplasm versus the nucleus [19], was found to interact with the anti-senescence proteins ID1 and E2F1 and the anti-stress proteins FAK and HIF1a, causing them to be retained in the cytoplasm, preventing their nuclear translocation to inhibit their antisenescence and anti-stress functions [42]. Another study by the same research group showed that circFoxo3 is able to bind to the cell cycle proteins cyclin-dependent kinase 2 (CDK2) and cyclin-dependent kinase inhibitor 1 (p21), resulting in the formation of a circ-Foxo3-p21-CDK2 ternary complex, suppressing the cell cycle and blocking the transition from G1 to S phase [18]. Other studies further proved circRNAs bind and sequester proteins. Ashwal-Fluss et al. proved that circMbl can sequester excess MBL to regulate the production balance of the mbl and circMbl [34]. Abdelmohsen et al. proposed that the extensive binding of CircPABPN1 to HuR prevents HuR binding to PABPN1 mRNA and lowers PABPN1 translation [43].

Consider the examples above, we could put forward an interesting possibility that ceRNAs including lncRNAs, circRNAs as well as other molecules may interact with each other with RNA binding protein shared response elements in the cytoplasm.

#### Regulating gene transcription

Although most circRNAs regulate miRNAs as the role of miRNA sponge, some circular RNAs could cis or trans regulate gene transcription (Fig. 2H). Researchers found that, knockdown of ciRNAs, which exists in the nucleus and have little enrichment for microRNA target sites led to the reduced expression of their parent genes. Importantly, ci-ankrd52 and ci-sirt7 can function as positive regulators of their parental gene transcription by interacting with Pol II [27], which indicating that intron circular RNAs can regulate parental gene transcription. Exon-intron circRNAs (EIciRNAs), have also been shown to function as transcription factors. Li et al. found that both of the two exon-intron circular RNAs, circ-EIF3J and circ-PAIP2, can combine with the U1 small nuclear ribonucleic proteins (snRNPs) to further interact with RNA Polymerase II in the promoter region of the host gene to enhance the expression of their parental genes in HeLa and HEK293 cells [28]. As a result, EIciRNAs can play an important role in positive feedback regulation. In addition, the ecRNA, circANRIL, may reduce the content of ANRIL protein, regulating the expression of INK4/ARF [44]. In summary, circRNA could regulate gene expression by combining RNA polymerase II complex and transcripting related proteins.

#### Translation

Due to without 5′–3′ polarity and polyadenylated tail as well as lacking internal ribosome entry site (IRES), most researchers believed that circRNAs were a distinct class of endogenous noncoding RNAs that could not translate proteins. However, considering that most circRNAs are generated from coding genes and contain complete exons, a few circRNAs were proven to have the potential to be translated into proteins (Fig. 2I). Studies of Chen et al. have shown that after inserting an internal ribosome entry site (IRES) into a synthetic circRNA, the eukaryotic ribosomal 40S subunit would bind to circRNAs at the IRES and initiate the translation process, both in vitro and in vivo [45]. Also, green fluorescent protein (GFP) can produce extremely long protein chains by transfecting an artificial circRNA inserted with a GFP open reading frame in *Escherichia coli* [46]. Importantly, viral circRNA is known to encode proteins in eukaryotic cells. For example, circRNAs in hepatitis D virus (HDV) could encode the hepatitis D virus antigen (HDAg) after infecting eukaryotic cells [47]. Additionally, A recent study of Legnini I. et al. found that circ-ZNF609 could translate proteins in murine myoblasts when driven by IRES [48]. Pamudurti N. R. et al. found circMbl3 could translate protein in fly heads [49]. In summary, more and more evidence proved that circRNAs could translate proteins directly. However, these discoveries showed that notion of circRNAs being non-coding RNAs is doubtful.

## CircRNA and Breast Cancer

Early studies showed that circRNAs are differentially expressed in many cancerous tissues and massive circRNAs have been discovered in tumorous tissue with the development of high-throughput sequencing, including in breast cancer, suggesting that circRNAs may be exploited for diagnostic and therapeutic applications.

### Expression of circRNA in breast cancer

Accumulating evidence has revealed that higher circRNA numbers were detected in normal breast mammary tissues than the tumor tissues. By means of circRNA microarray analysis, Yin et al. found that 41 circRNAs were differently expressed with 2 fold change in the plasma of breast cancer patients compared to healthy controls, including 19 up-regulated and 22 down-regulated [50]. Nair et al. [51] developed a Circ-Seq workflow to identified circRNAs specific to breast tumor samples and catalogued circRNAs unique to each of the three breast cancer subtypes: triple negative (TN), estrogen receptor positive (ER+), and ErbB2 overexpressed-HER2 positive (HER2+). Notably, a lower number of circRNAs were observed in breast tumors compared to both normal-adjacent breast tissues from TCGA (The Cancer Genome Atlas) as well as normal mammary tissue samples from GTEx (Genotype-Tissue Expression). What’s more, circRNAs in estrogen receptor (ER) positive normal-adjacent samples is inversely correlated to the risk-of-relapse proliferation (ROR-P) score for proliferating genes, suggesting that circRNAs may be markers of cell proliferation in breast cancer and associate with cancer subtypes. In a recent study, researchers screened the circRNA expression profiles in breast cancer and adjacent normal-appearing tissues using circRNA microarray analysis [52]. The results showed that 1155 circRNAs were differentially expressed, among which 715 were upregulated and 440 were downregulated in breast cancer tissues. Then the validation study demonstrated that hsa_circ_103110, hsa_circ_104689 and hsa_circ_104821 levels were elevated in breast cancer tissues, whereas hsa_circ_006054, hsa_circ_100219 and hsa_circ_406697 were downregulated. In a similar way, Tang et al. discovered totally 1705 circRNAs were identified to be significantly aberrant in breast cancer tissue using circRNA microarray analysis [53]. Zhang et al. [54] found that 1314 circRNAs were detected at both lactation stages in lactating rats. In addition, four protein-coding genes (Rev3l, IGSF11, MAML2, and LPP) that also transcribe high levels of circRNAs have been reported to be involved in cancer. With a growing number of circRNAs have been discovered in breast cancer, the role of circRNA will get more attention. CircRNAs that have been found related with breast cancer are shown in Table 1.

**Table 1:** Circular RNA in breast cancer

Name	Dysregulation	Sponge target	Funcion	Ref
hsa_circ_0001982	upregulated	miR-143	Oncogene	[53]
circGFRA1	upregulated	miR-34a	Oncogene/biomarker	[59]
circ-ABCB10	upregulated	miR-1271	Oncogene	[57]
hsa_circ_103110	upregulated	–	Biomarker	[52]
hsa_circ_104821	upregulated	–	Biomarker	[52]
hsa_circ_104689	upregulated	–	Biomarker	[52]
hsa_circ_006054	downregulated	–	Biomarker	[52]
hsa_circ_100219	downregulated	–	Biomarker	[52]
hsa_circ_406697	downregulated	–	Biomarker	[52]
circ-Foxo3	downregulated	MiR-22/miR-136/miR-138	Tumor suppressor	[71]
CircRNA_1093	upregulated	MiR-342-3p	Oncogene	[54]
circVRK1	downregulated	–	Tumor suppressor	[81]
hsa_circ_0001785	upregulated	–	Biomarker	[50]
hsa_circ_0108942	upregulated	–	Biomarker	[50]
hsa_circ_0068033	downregulated		Biomarker	[50]

### CircRNA and the proliferation and progression of breast cancer

#### circRNA regulates the proliferation and progression of breast cancer via serving as microRNA sponges

Accumulating evidence have proved that miRNAs regulate gene expression in most biological processes, including in carcinogenesis. In depth study revealed that some circRNAs may function as microRNA sponges in regulating the proliferation, metastasis and invasion of cancer. For example, some studies have shown the essential role of miR-124-3p as a tumor suppressor in breast tumorigenesis [55]. CircHIPK3, an abundant circRNA derived from Exon2 of the HIPK3 gene, was observed to sponge to 9 miRNAs with 18 potential binding sites via a luciferase screening assay. Specifically, they show that circHIPK3 binds to miR-124 directly and inhibits miR-124 activity, inducing the proliferation of breast cancer [56]. The study of Tang et al. [53] reveals that the circRNA, hsa_circ_0001982, is markedly overexpressed in breast cancer tissue and cell lines. Furtherly, they performed loss-of-function and rescue experiments to investigate the biological and miRNA ‘sponge’ functions of hsa_circ_0001982 in the tumorigenesis and progression. Results revealed that hsa_circ_0001982 knockdown suppressed breast cancer cell proliferation and invasion and induced apoptosis by targeting miR-143, providing a novel insight for breast cancer pathogenesis. In a similar way, results of another study discovered a significantly up-regulated circRNA hsa_circ_0008717 in breast cancer tissue and they nominate it as circ-ABCB10 [57]. In follow-up RT-PCR validation, the significant up-regulation of circ-ABCB10 was confirmed in a larger sample size and cell line. In vitro, loss-of-function experiments showed circ-ABCB10 knockdown suppressed the proliferation and increased apoptosis of breast cancer cells, revealing the important regulatory role of circ-ABCB10 through sponging miR-1271 [57]. CircGFRA1 has been reported upregulated in breast cancer and was positively correlated with tumor size, TNM staging, lymph node metastasis and histological grade of TNBC [58]. Further experiments showed that circGFRA1 could promote proliferation and inhibit apoptosis in TNBC. Researchers proposed that circGFRA1 might function as miR-34a sponge to regulate GFRA1 expression through the ceRNA mechanism [59]. Researches above establishe a novel approach whereby circRNAs can regulate the progression of cancer through sequestering specific miRNA species associated with proliferation, differentiation, migration and carcinogenesis process.

#### CircRNA regulates the proliferation and progression of breast cancer via cancer-associated signaling pathways

**Fig. 3:**
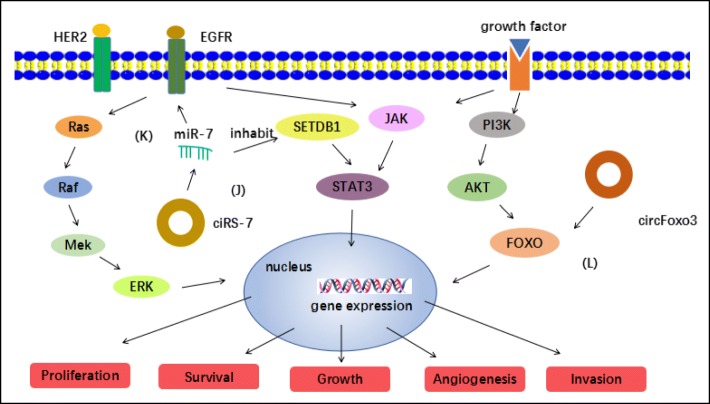
CircRNA regulates the proliferation and progression of breast cancer via cancer-associated signaling pathways: (J) ciRS -7 could act as a miR-7 inhibitor to weaken the suppression of miR-7 to STAT3 pathway; (K) ciRS -7 could participate in the activation of EGFR pathway via inhibiting miR-7; (L) circ-Foxo3 can up-regulate Foxo3 protein levels by binding to a number of microRNAs shared with the Foxo3 linear mRNA, inducing cell apoptosis and inhibit progression of breast cancer through PI3K/AKT/FOXO pathway

Cell proliferation in breast cancer are involved in the aberrant activation of multiple cancer-associated signaling pathways. An increasing amount of evidence has demonstrated the relationship between various novel circRNAs and signaling pathways with carcinogenesis. Increasing evidence have suggested that circRNAs play an important role in the initiation, proliferation, metastasis and invasion of breast cancer by regulating the target genes directly or interacting with miRNAs closely associated with cancer-related signaling pathways. For example, Huang et al. have found that circ-ITCH plays an inhibitory role in colorectal cancer by regulating the Wnt/beta-catenin pathway [60]. Similarly, a recent study manifested that the inactivation of the hsa_circ_0011946/RFC3 signaling pathway could inhibit the migration and invasion capacities of MCF-7 cells [61]. As a tumor suppressor miRNA [62], miR-7 expression is decreased in malignant than normal breast tissue and forced expression of miR-7 in aggressive breast cancer cell lines suppressed tumor cell proliferation, migration and invasion [63]. Up to date, miR-7 has been proved involved in many cancer- associated signaling pathways via directly down-regulating expression of crucial oncogenic factors such as EGFR [64], FAK [63], KLF4 [65], HER2D16 [66], REGc [67], SETDB1 [68] and so on, indicating a clear tumor-suppressive role for miR-7. As mentioned above, the newly identified ciRS-7, as a circular miR-7 inhibitor, is known to be involved in many cancer-associated signaling pathways. Zhang et al. proved that miR-7, which was downregulated in breast CSCs (BCSCs) isolated from the human MCF-7 and MDA-MB-231 cell lines, inhibited cell invasion and metastasis, decreased the BCSC population and partially reversed epithelial-to-mesenchymal transition (EMT) in MDA-MB-231 cells by directly targeting the oncogene, SETDB1 and furtherly, resulting in the suppression of STAT3. Thus, ciRS -7 may act as a miR-7 inhibitor to weaken the suppression of miR-7 to STAT3 pathway [68] (Fig. 3J). CiRS-7 promotes cell proliferation in breast cancer by MiR-7 inhibiting MCF-7/HER2Δ16 cell migration through a mechanism involving suppression of the miR-7 target gene EGFR. Namely, ciRS -7 could participate in the activation of EGFR pathway via inhibiting miR-7 [66] (Fig. 3K). Researches above indicates that circRNA might induce the proliferation and progression of breast cancer via cancer-associated signaling pathways. However, some evidence shows the opposite point of view. PI3K/AKT/FOXO pathway has been proved involved in breast cancer tumor initiation via the integrated genomic approach [69]. Foxo3 has been classified as a tumour suppressor gene due to the down-regulation of Foxo3 expression in cancer development due to increased Akt activity or loss of PTEN [70]. Yang et al. showed that circ-Foxo3 can up-regulate Foxo3 protein levels by binding to a number of microRNAs shared with the Foxo3 linear mRNA [71], inducing cell apoptosis and inhibit progression of breast cancer through PI3K/AKT/FOXO pathway (Fig. 3L). We believe that increasing circRNAs will be investigated to participate in initiation of breast cancer via cancer-associated signaling pathways.

### CircRNA as potential diagnostic and prognostic biomarkers in breast cancer

It is well known that most cancer types can be cured, if diagnosed at an early stage. The molecular pathogenesis of breast cancer is complex and shows heterogeneous. Considering the currently used cancer diagnostic markers including CT, MRI and histopathology are invasive or expensive, minimally invasive and inexpensive methods are imperatively required. Besides, prognostic evaluation also plays a significance role in early intervention of poor prognostic factors as well as the prolongation of the life expectancy of cancer patients. Recent studies have shown that circRNAs are involved in multiple pathological processes of breast cancer. The clinical potential for use of circRNAs as diagnostic and prognostic biomarkers for breast cancer is increasingly being investigated. According to the current studies, the main characteristics of circRNAs are as the following: (1) Diversity: more than 100,000 circRNAs have been identified in human tissues detected by high-throughput sequencing [72]. (2) Highly abundant expression: circRNAs comprise over 14% of the transcribed genes in fibroblasts. Although the overall abundance of circRNAs is low, the expression of some circRNAs is much higher than that of linear RNAs [31]. (3) Stability: without 5′-3′ polarity and polyadenylated tail, circRNAs are more stable than liner RNA and can resist to degradation by RNA exonuclease or RNase R [73]. In addition, the half-life of circRNAs in most species is longer than 48 h, while the average half-life of mRNAs is 10 h [74]. (4) Specificity: circRNAs show cell type-specific and tissue-specific expression [75]. (5) Universality: a report in 2012 confirmed that circRNAs are the most common molecules after linear RNAs in human cells [76]. (6) Conservatism: the signal behind circularization seems to be evolutionarily conserved in different species [77]. Hence the properties above confer distinct advantages to circRNAs as potential biomarkers of cancer diagnosis and prognosis in breast cancer.

For example, the plasma levels of hsa_circ_0001785 in post-operative patients were significantly decreased compared to pre-operative patients [50], showing the important role to be a prognosis biomarker. This phenomenon might be mainly due to the decreasing release of tumor-derived nucleic acid after mammary tumor excision [78]. Besides, hsa_circ_0001785 plasma level was closely related to histological grade, TNM stage and distant metastasis, which helps to the staging and grading of breast cancer. What’s more, hsa_circ_0001982 [53] and circ-ABCB10 [57] knockdown suppressed the proliferation and increased apoptosis of breast cancer cells, CircGFRA1 has been reported upregulated in breast cancer and was positively correlated with tumor size, TNM staging, lymph node metastasis and histological grade of TNBC [59]. The above examples are only a fraction of the several instances in which circRNAs have demonstrated promising diagnostic and prognostic potential in breast cancer. However, more studies are required before circRNAs can be recommended for human use.

### Therapeutic potential of circRNAs in breast cancer

#### CircRNAs exhibit potential anti-cancer effects

Currently, a number of circRNAs have been proved associated with proliferation and progression of breast cancer, manifesting their potential roles as targets in breast cancer therapy. The recent study of Wang et al. reveal that circRNA-000911 plays an anti-oncogenic role in breast cancer by serving as a miRNA sponge for miR-449a and thereby promoting the function of Notch1 and the NF-κB signaling pathway [79]. Therefore, the overexpression of circRNA-000911 may provide a future direction which may aid in the development of a novel treatment strategy for breast cancer. The capacity of circ-Foxo3 in inducing cell apoptosis and inhibit progression of breast cancer makes it a promising therapeutic target of breast cancer [71]. Currently, accumulating evidence suggests that breast cancer departs from a fraction of cancer initiating cells called cancer stem cells (CSCs) [80], which are responsible for metastasis and recurrence of the tumor. Conventional therapies fail eventually owing to not killing CSCs, which results in recurrence of tumors. To prevent breast cancer recurrence and metastasis, it will be crucial to eradicate BCSC. In a recent study, Yan et al. screened the circRNA profile in breast cancer stem cells (BCSCs) using RNA-Sequencing. 27 circRNAs were found to be aberrantly expressed of which 19 circRNAs were downregulated and 8 were upregulated. Importantly, they found that circular RNA VRK1 (circVRK1) could suppress BCSC’s expansion and self-renewal capacity. These findings indicate that circVRK1 might be a promising target for BCSCs [81].

#### Relationship between circRNA and chemoresistant breast cancer

Chemotherapy is an effective strategy for the clinical treatment of breast cancer but sometimes the efficacy of chemotherapeutics is reduced due to drug resistance, which correlates with treatment failure and poor prognosis among breast cancer patients. It is vital to understand the molecular pathways involved in the pathogenesis of breast cancer that cause metastasis and chemotherapy resistance. For example, Adriamycin (ADM) treatment is facing the challenging. Gao et al. [82] detected 3093 circRNAs and identified 18 circRNAs that were differentially expressed in ADMresistant MCF-7 human breast cancer cells (MCF-7/ADM) compared with parental MCF-7 cells. Importantly, a higher expression level of hsa_circ_00006528 was observed in the ADM-resistant cell lines and tissues than in the ADM-sensitive groups. In addition, a regulatory role of the hsa_circ_00006528-miR-7–5p-Raf1 axis in ADM-resistant breast cancer was identified, suggesting that hsa_circ_00006528 expression is significantly associated with ADM-resistant breast cancers and demonstrate the potential function of hsa_circ_00006528 in overcoming drug resistance. In addition, Miao et al. proved that miR-130b targets PTEN to induce multidrug resistance (MDR), proliferation and apoptosis via PI3K/Akt signaling pathway [83]. Regarding the mechanism of circRNAs, we suspected that certain circRNAs could act as sponges for hsa-miR-130b, and several target genes related to PI3K/Akt signaling pathways, participating in chemotherapeutic resistance of breast cancer. Considering certain circRNAs are associated with chemoresistant breast cancer, dynamic analysis of aberrantly expressed circRNAs in different sensitivities to a specific chemotherapy treatment is very crucial for further therapy in breast cancer.

#### Breast cancer therapy targeting circRNAs

It has been shown that certain circRNAs are involved in various biological processes of breast cancer, including proliferation, migration, invasion, and apoptosis. Hence, Therapeutic strategies targeting circRNAs is expected to provide a new viewpoint for breast cancer therapy. Currently, several technologies offer an unprecedented opportunity for partial or complete removal of oncogenic circRNA, including siRNA-based therapy [84], anti-sense oligonucleotides therapy [85], CRISPER/Cas system [86] and so on. On the contrary, previously researches suggest that miRNAs involved in cancer can be divided into oncomiRs and tumor suppressor miRNAs. OncomiRs, as harmful miRNAs, could be countered by circRNAs via the function of miRNA sponges or other pathways. Since certain circRNA has many binding sites for a specific miRNA, it is more effective than typical miRNA inhibitors. The permuted intron-exon (PIE) method is a promising methodology for producing circRNA drugs [87]. Such synthetic circRNA inhibitors might be future targets for therapies of breast cancer [88]. How to deliver circRNAs efficiently to the accurate action site? Previous studies present that extracellular vesicles (EVs) are likely to be engineered to deliver circRNAs efficiently to a target tissue [89].

## Research strategies for circRNAs

**Fig. 4:**
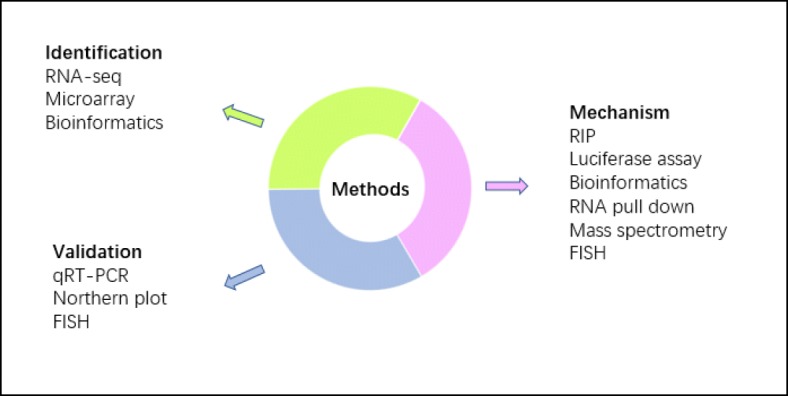
The Research strategies for circRNAs

Ongoing research is furthering our understanding of the complex circRNA network. Various methods have been developed to detect circRNA expression and investigate their functions (Fig. 4). High-throughput RNA sequencing (RNA-seq) and microarray in human cells, which are widely used technology to annotate new RNA species and quantify RNA abundance, have identified the majority of circRNAs. In addition, a number of bioinformatic algorithms have been developed for identifying circRNAs. However, it should be noted that there are always shortages regardless of the algorithm used. Hansen et al. [90] use common RNA-seq datasets to scrutinize and compare the output from five different algorithms (circRNA_finder, find_circ, CIRCexplorer, CIRI, and MapSplice) and evaluate the levels of bona fide and false positive circRNAs based on RNase R resistance. By this approach, they observe that CIRCexplorer and Mapsplice showed the best accuracy and good sensitivity with respect to circRNA detection but the processing speed is low. The others showed the comparatively opposite outcome. Herein, it is advisable to run more than one circRNA algorithms to achieve reliable predictions. Quantitative real-time PCR (qRT-PCR) can be used as validation methods for circRNA expression. Northern blotting is a more stringent circRNA validation method than qRT-PCR as no reverse transcription and amplification steps are part of the protocol [91]. Fluorescence in situ hybridization (FISH) could provide spatial information about certain circRNAs [92]. To reveal circRNA-miRNA and circRNA-RBP interactions, AGO2 RNA immunoprecipitation (RIP), luciferase reporter assay, bioinformatic prediction and RNA pull down combined with mass spectrometry are suggested methods [93, 94].

## Online databases

Several databases and web tools have been constructed to provide the basic information about circRNAs and their potential regulatory networks (Table 2). Cirbase, a comprehensive database providing online public circRNA datasets, can be used to download the sequence of interested circRNA. In addition, the position of the circRNAs in the genome and its expression pattern in various tissues and cells can be accessed. A similar database is Circ2Traits which mainly focuses on circRNA potentially associated with disease and traits. Circnet provides circRNA-miRNA-gene regulatory networks which will facilitate further research on circRNAs. CircInteractome (circRNA interactome) is a web-based tool for designing primers for specific detection of circRNAs of interest and their interacting microRNAs and proteins. CIRCpedia aims to annotating alternative back-splicing and alternative splicing in circRNAs across different cell lines. CircRNADb includes detailed information of the circRNA like genomic information, exon splicing, genome sequence, IRES, and open reading frames (ORFs). TSCD is a database of tissue-specific circular RNAs in the human and mouse genomes. CSCD, a cancer-specific circRNA database has also been constructed. With the help of these online databases, differential expression of circRNAs between tissue and cells could be analyzed and the role of circRNA in physiological and pathological processes could be further explored.

**Table 2:** Online circRNA databases and webtools

Name	Description	Website	Ref
circBase	A comprehensive database for public circRNA datasets	www.circbase.org	[72]
circ2Traits	A knowledgebase of human circRNAs associated with diseases or traits	gyanxet-b.com/circdb	[95]
Circnet	A database of circular RNAs derived from transcriptome sequencing data	circnet.mbc.nctu.edu.tw	[96]
CircInteractome	Exploring circRNAs and their interaction with proteins or miRNAs, as well as primer design and siRNA design	circinteractome.nia.nih.gov	[97]
CIRCpedia	Annotating alternative back-splicing and alternative splicing in circRNAs	www.picb.ac.cn/rnomics/circpedia	[30]
circRNADb	A comprehensive database for human circular RNAs with protein-coding annotations a database for cancer-specific	reprod.njmu.edu.cn/circrnadb	[98]
CSCD	circular RNAs A database of tissue-specific	gb.whu.edu.cn/CSCD	[99]
TSCD	circular RNAs in the human and mouse genomes	gb.whu.edu.cn/TSCD	[100]

## Conclusions and Perspective

CircRNAs, which were thought to be errors in RNA splicing, are now regarded as an emerging key player with intriguing molecular functions in the RNA world. The abundant and stable class of RNA molecules with a range of functions, including sponge, regulation molecules, translation, biomarker and tumor suppressor are regarded as vital regulators of various physiological and pathophysiological processes. Recently, many studies have explored the clinical values of circRNAs in cancers. As summarized in this review, circRNAs are involved in various biological processes of breast cancer, including proliferation, migration, invasion, and apoptosis. As promising biomarkers in breast cancer diagnosis, prognosis, recurrence and risk evaluation, circRNAs are potential therapeutic targets. In conclusion, circRNAs provide a new perspective for the diagnosis and treatment of breast cancer. Nevertheless, compared with coding RNA and miRNA and lncRNA, there are still significant gaps in our current understanding of circRNAs and we are still far being able to incorporate circRNA into clinical practice. Here are some recommendations for future research of circRNAs in breast cancer. First, despite a growing number of papers on circRNAs have been published, conclusive answers about the precise mechanisms of circRNA underlying the initiation and progression of cancer are worth an indepth study. Second, currently the detection of circRNA in cancer mainly focus on tissue samples which is invasive and experience. Non-invasive clinical samples (blood, urine, saliva, etc.) and samples related to the disease (gastric juice, cerebrospinal fluid, etc.) should be tested for circRNA expression in the future research. Third, certain circRNAs have been verified as potential diagnostic and prognosis biomarkers but their precesion need further research. Combined detection including the combined of different circRNAs and the combined of circRNAs and traditional biomarkers deserved to be considered. Fourth, considering the important roles of circRNAs as potential targets for cancer therapy, how to deliver circRNAs efficiently to the accurate action site with a long-term sustained effect and without immunological rejection are imperatively to be studied. Fifth, since the ultimate goal of circRNA-related studies is applying cancer specific circRNAs to human diseases safely, more controlled clinical studies comprising a large number of patients are required beforehand.

With the development of research methods for identifying and validating novel circRNAs and the help of online public databases, we believe that one day, the appropriate and precise use of circRNAs in clinical diagnosis and treatment will bring tremendous progress for cancer therapy.

## References

[1] Siegel RL, Miller KD, Jemal A (2017). Cancer statistics, 2017[J]. CA Cancer J Clin.

[2] Guo Y, Warren Andersen S, Shu XO, Michailidou K, Bolla MK, Wang Q (2016). Genetically predicted body mass index and breast Cancer risk: Mendelian randomization analyses of data from 145,000 women of European descent [J]. PLoS Med.

[3] Chlebowski RT, Manson JE, Anderson GL, Cauley JA, Aragaki AK, Stefanick ML (2013). Estrogen plus progestin and breast cancer incidence and mortality in the Women's Health Initiative observational study [J]. J Natl Cancer Inst.

[4] Lambertini M, Santoro L, Del Mastro L, Nguyen B, Livraghi L, Ugolini D (2016). Reproductive behaviors and risk of developing breast cancer according to tumor subtype: a systematic review and meta-analysis of epidemiological studies [J]. Cancer Treat Rev.

[5] Rice MS, Eliassen AH, Hankinson SE, Lenart EB, Willett WC, Tamimi RM (2016). Breast Cancer research in the Nurses' health studies: exposures across the life course [J]. Am J Public Health.

[6] Wu L, Shen Y, Peng X, Zhang S, Wang M, Xu G (2016). Aberrant promoter methylation of cancer-related genes in human breast cancer [J]. Oncol Lett.

[7] Karsli-Ceppioglu S, Dagdemir A, Judes G, Ngollo M, Penault-Llorca F, Pajon A (2014). Epigenetic mechanisms of breast cancer: an update of the current knowledge [J]. Epigenomics.

[8] Mulrane L, McGee SF, Gallagher WM, O'Connor DP (2013). miRNA dysregulation in breast cancer [J]. Cancer Res.

[9] Sidiropoulos KG, Ding Q, Pampalakis G, White NM, Boulos P, Sotiropoulou G (2016). KLK6-regulated miRNA networks activate oncogenic pathways in breast cancer subtypes [J]. Mol Oncol.

[10] Memczak S, Jens M, Elefsinioti A, Torti F, Krueger J, Rybak A (2013). Circular RNAs are a large class of animal RNAs with regulatory potency [J]. Nature.

[11] Sanger HL, Klotz G, Riesner D, Gross HJ, Kleinschmidt AK (1976). Viroids are single-stranded covalently closed circular RNA molecules existing as highly base-paired rod-like structures [J]. Proc Natl Acad Sci U S A.

[12] Hsu MT, Coca-Prados M (1979). Electron microscopic evidence for the circular form of RNA in the cytoplasm of eukaryotic cells [J]. Nature.

[13] Guo JU, Agarwal V, Guo H, Bartel DP (2014). Expanded identification and characterization of mammalian circular RNAs [J]. Genome Biol.

[14] Chen LL, Yang L (2015). Regulation of circRNA biogenesis [J]. RNA Biol.

[15] Cocquerelle C, Mascrez B, Hetuin D, Bailleul B (1993). Mis-splicing yields circular RNA molecules [J]. FASEB journal: official publication of the Federation of American Societies for Experimental Biology.

[16] Lukiw WJ. Circular RNA (circRNA) in Alzheimer's disease (AD)[J]. Front Genet 2013,4:307. 10.3389/fgene.2013.00307 PMC3875874 24427167

[17] Wang K, Long B, Liu F, Wang JX, Liu CY, Zhao B (2016). A circular RNA protects the heart from pathological hypertrophy and heart failure by targeting miR-223[J]. Eur Heart J.

[18] Du WW, Yang W, Liu E, Yang Z, Dhaliwal P, Yang BB (2016). Foxo3 circular RNA retards cell cycle progression via forming ternary complexes with p21 and CDK2[J]. Nucleic Acids Res.

[19] Guarnerio J, Bezzi M, Jeong JC, Paffenholz SV, Berry K, Naldini MM (2016). Oncogenic role of fusion-circRNAs derived from Cancer-associated chromosomal translocations [J]. Cell.

[20] Bahn JH, Zhang Q, Li F, Chan TM, Lin X, Kim Y (2015). The landscape of microRNA, Piwi-interacting RNA, and circular RNA in human saliva [J]. Clin Chem.

[21] Memczak S, Papavasileiou P, Peters O, Rajewsky N (2015). Identification and characterization of circular RNAs as a new class of putative biomarkers in human blood [J]. PLoS One.

[22] Szabo L, Morey R, Palpant NJ, Wang PL, Afari N, Jiang C (2015). Statistically based splicing detection reveals neural enrichment and tissue-specific induction of circular RNA during human fetal development [J]. Genome Biol.

[23] Qin M, Liu G, Huo X, Tao X, Sun X, Ge Z (2016). Hsa_circ_0001649: A circular RNA and potential novel biomarker for hepatocellular carcinoma [J]. Cancer biomarkers: section A of Disease markers.

[24] Li P, Chen S, Chen H, Mo X, Li T, Shao Y, et al. Using circular RNA as a novel type of biomarker in the screening of gastric cancer [J]. Clinica chimica acta; international journal of clinical chemistry. 2015,444:132–6. 10.1016/j.cca.2015.02.018 25689795

[25] Chen I, Chen CY, Chuang TJ (2015). Biogenesis, identification, and function of exonic circular RNAs [J]. Wiley interdisciplinary reviews RNA.

[26] Zhang XO, Wang HB, Zhang Y, Lu X, Chen LL, Yang L (2014). Complementary sequence-mediated exon circularization [J]. Cell.

[27] Zhang Y, Zhang XO, Chen T, Xiang JF, Yin QF, Xing YH (2013). Circular intronic long noncoding RNAs [J]. Mol Cell.

[28] Li Z, Huang C, Bao C, Chen L, Lin M, Wang X (2015). Exon-intron circular RNAs regulate transcription in the nucleus [J]. Nat Struct Mol Biol.

[29] Westholm JO, Miura P, Olson S, Shenker S, Joseph B, Sanfilippo P (2014). Genome-wide analysis of drosophila circular RNAs reveals their structural and sequence properties and age-dependent neural accumulation [J]. Cell Rep.

[30] Zhang XO, Dong R, Zhang Y, Zhang JL, Luo Z, Zhang J (2016). Diverse alternative back-splicing and alternative splicing landscape of circular RNAs [J]. Genome Res.

[31] Jeck WR, Sorrentino JA, Wang K, Slevin MK, Burd CE, Liu J, et al. Circular RNAs are abundant, conserved, and associated with ALU repeats [J]. RNA (New York, NY). 2013,19(2):141–57. 10.1261/rna.035667.112 PMC3543092 23249747

[32] Schmidt CA, Noto JJ, Filonov GS, Matera AG (2016). A method for expressing and imaging abundant, stable, circular RNAs in vivo using tRNA splicing [J]. Methods Enzymol.

[33] Lu Z, Filonov GS, Noto JJ, Schmidt CA, Hatkevich TL, Wen Y, et al. Metazoan tRNA introns generate stable circular RNAs in vivo [J]. RNA (New York, NY). 2015,21(9):1554–65. 10.1261/rna.052944.115 PMC4536317 26194134

[34] Ashwal-Fluss R, Meyer M, Pamudurti NR, Ivanov A, Bartok O, Hanan M (2014). circRNA biogenesis competes with pre-mRNA splicing [J]. Mol Cell.

[35] Rybak-Wolf A, Stottmeister C, Glazar P, Jens M, Pino N, Giusti S (2015). Circular RNAs in the mammalian brain are highly abundant, conserved, and dynamically expressed [J]. Mol Cell.

[36] Conn SJ, Pillman KA, Toubia J, Conn VM, Salmanidis M, Phillips CA (2015). The RNA binding protein quaking regulates formation of circRNAs [J]. Cell.

[37] Bartel DP (2009). MicroRNAs: target recognition and regulatory functions [J]. Cell.

[38] Tay Y, Rinn J, Pandolfi PP (2014). The multilayered complexity of ceRNA crosstalk and competition [J]. Nature.

[39] Hansen TB, Jensen TI, Clausen BH, Bramsen JB, Finsen B, Damgaard CK (2013). Natural RNA circles function as efficient microRNA sponges [J]. Nature.

[40] Li F, Zhang L, Li W, Deng J, Zheng J, An M (2015). Circular RNA ITCH has inhibitory effect on ESCC by suppressing the Wnt/beta-catenin pathway [J]. Oncotarget.

[41] Militello G, Weirick T, John D, Doring C, Dimmeler S, Uchida S (2017). Screening and validation of lncRNAs and circRNAs as miRNA sponges [J]. Brief Bioinform.

[42] Du WW, Yang W, Chen Y, Wu ZK, Foster FS, Yang Z (2017). Foxo3 circular RNA promotes cardiac senescence by modulating multiple factors associated with stress and senescence responses [J]. Eur Heart J.

[43] Abdelmohsen K, Panda AC, Munk R, Grammatikakis I, Dudekula DB, De S (2017). Identification of HuR target circular RNAs uncovers suppression of PABPN1 translation by CircPABPN1[J]. RNA Biol.

[44] Burd CE, Jeck WR, Liu Y, Sanoff HK, Wang Z, Sharpless NE (2010). Expression of linear and novel circular forms of an INK4/ARF-associated non-coding RNA correlates with atherosclerosis risk [J]. PLoS Genet.

[45] Chen CY, Sarnow P. Initiation of protein synthesis by the eukaryotic translational apparatus on circular RNAs [J]. Science (New York, NY). 1995,268(5209):415–7. 10.1126/science.7536344 7536344

[46] Perriman R, Ares M, Jr. Circular mRNA can direct translation of extremely long repeating-sequence proteins in vivo [J]. RNA (New York, NY). 1998,4(9):1047–54. 10.1017/s135583829898061x PMC1369681 9740124

[47] Kos A, Dijkema R, Arnberg AC, van der Meide PH, Schellekens H (1986). The hepatitis delta (delta) virus possesses a circular RNA [J]. Nature.

[48] Legnini I, Di Timoteo G, Rossi F, Morlando M, Briganti F, Sthandier O, et al. Circ-ZNF609 Is a Circular RNA that Can Be Translated and Functions in Myogenesis [J]. Molecular cell. 2017,66(1):22–37.e9. 10.1016/j.molcel.2017.02.017 PMC5387670 28344082

[49] Pamudurti NR, Bartok O, Jens M, Ashwal-Fluss R, Stottmeister C, Ruhe L, et al. Translation of CircRNAs [J]. Molecular cell. 2017,66(1):9–21.e7. 10.1016/j.molcel.2017.02.021 PMC5387669 28344080

[50] Yin WB, Yan MG, Fang X, Guo JJ, Xiong W, Zhang RP. Circulating circular RNA hsa_circ_0001785 acts as a diagnostic biomarker for breast cancer detection [J]. Clinica chimica acta; international journal of clinical chemistry. 2017. 10.1016/j.cca.2017.10.011 29045858

[51] Nair AA, Niu N, Tang X, Thompson KJ, Wang L, Kocher JP (2016). Circular RNAs and their associations with breast cancer subtypes [J]. Oncotarget.

[52] Lu L, Sun J, Shi P, Kong W, Xu K, He B (2017). Identification of circular RNAs as a promising new class of diagnostic biomarkers for human breast cancer [J]. Oncotarget.

[53] Tang YY, Zhao P, Zou TN, Duan JJ, Zhi R, Yang SY (2017). Circular RNA hsa_circ_0001982 promotes breast Cancer cell carcinogenesis through decreasing miR-143[J]. DNA Cell Biol.

[54] Zhang C, Wu H, Wang Y, Zhao Y, Fang X, Chen C (2015). Expression patterns of circular RNAs from primary kinase transcripts in the mammary glands of lactating rats [J]. J Breast Cancer.

[55] Wang Y, Chen L, Wu Z, Wang M, Jin F, Wang N, et al. miR-124-3p functions as a tumor suppressor in breast cancer by targeting CBL [J]. BMC cancer. 2016,16(1):826. 10.1186/s12885-016-2862-4 PMC5109743 27842510

[56] Zheng Q, Bao C, Guo W, Li S, Chen J, Chen B (2016). Circular RNA profiling reveals an abundant circHIPK3 that regulates cell growth by sponging multiple miRNAs [J]. Nat Commun.

[57] Liang HF, Zhang XZ, Liu BG, Jia GT, Li WL (2017). Circular RNA circ-ABCB10 promotes breast cancer proliferation and progression through sponging miR-1271[J]. Am J Cancer Res.

[58] Esseghir S, Todd SK, Hunt T, Poulsom R, Plaza-Menacho I, Reis-Filho JS (2007). A role for glial cell derived neurotrophic factor induced expression by inflammatory cytokines and RET/GFR alpha 1 receptor up-regulation in breast cancer [J]. Cancer Res.

[59] He R, Liu P, Xie X, Zhou Y, Liao Q, Xiong W, et al. circGFRA1 and GFRA1 act as ceRNAs in triple negative breast cancer by regulating miR-34a[J]. Journal of experimental & clinical cancer research: CR. 2017,36(1):145. 10.1186/s13046-017-0614-1 PMC5644184 29037220

[60] Huang G, Zhu H, Shi Y, Wu W, Cai H, Chen X. cir-ITCH plays an inhibitory role in colorectal cancer by regulating the Wnt/beta-catenin pathway [J]. PloS one. 2015,10(6):e0131225. 10.1371/journal.pone.0131225 PMC4482251 26110611

[61] Zhou J, Zhang WW, Peng F, Sun JY, He ZY, Wu SG (2018). Downregulation of hsa_circ_0011946 suppresses the migration and invasion of the breast cancer cell line MCF-7 by targeting RFC3[J]. Cancer Manag Res.

[62] Xin Z, Ma Q, Ren S, Wang G, Li F (2017). The understanding of circular RNAs as special triggers in carcinogenesis [J]. Briefings in functional genomics.

[63] Kong X, Li G, Yuan Y, He Y, Wu X, Zhang W (2012). MicroRNA-7 inhibits epithelial-to-mesenchymal transition and metastasis of breast cancer cells via targeting FAK expression [J]. PLoS One.

[64] Suto T, Yokobori T, Yajima R, Morita H, Fujii T, Yamaguchi S (2015). MicroRNA-7 expression in colorectal cancer is associated with poor prognosis and regulates cetuximab sensitivity via EGFR regulation [J]. Carcinogenesis.

[65] Okuda H, Xing F, Pandey PR, Sharma S, Watabe M, Pai SK (2013). miR-7 suppresses brain metastasis of breast cancer stem-like cells by modulating KLF4[J]. Cancer Res.

[66] Huynh FC, Jones FE (2014). MicroRNA-7 inhibits multiple oncogenic pathways to suppress HER2Delta16 mediated breast tumorigenesis and reverse trastuzumab resistance [J]. PLoS One.

[67] Shi Y, Luo X, Li P, Tan J, Wang X, Xiang T (2015). miR-7-5p suppresses cell proliferation and induces apoptosis of breast cancer cells mainly by targeting REGgamma [J]. Cancer Lett.

[68] Zhang H, Cai K, Wang J, Wang X, Cheng K, Shi F, et al. MiR-7, inhibited indirectly by lincRNA HOTAIR, directly inhibits SETDB1 and reverses the EMT of breast cancer stem cells by downregulating the STAT3 pathway [J]. Stem cells (Dayton, Ohio). 2014,32(11):2858–68. 10.1002/stem.1795 25070049

[69] Smit L, Berns K, Spence K, Ryder WD, Zeps N, Madiredjo M (2016). An integrated genomic approach identifies that the PI3K/AKT/FOXO pathway is involved in breast cancer tumor initiation [J]. Oncotarget.

[70] Du WW, Fang L, Yang W, Wu N, Awan FM, Yang Z (2017). Induction of tumor apoptosis through a circular RNA enhancing Foxo3 activity [J]. Cell Death Differ.

[71] Yang W, Du WW, Li X, Yee AJ, Yang BB (2016). Foxo3 activity promoted by non-coding effects of circular RNA and Foxo3 pseudogene in the inhibition of tumor growth and angiogenesis [J]. Oncogene.

[72] Glazar P, Papavasileiou P, Rajewsky N. circBase: a database for circular RNAs [J]. RNA (New York, NY). 2014,20(11):1666–70. 10.1261/rna.043687.113 PMC4201819 25234927

[73] Suzuki H, Zuo Y, Wang J, Zhang MQ, Malhotra A, Mayeda A (2006). Characterization of RNase R-digested cellular RNA source that consists of lariat and circular RNAs from pre-mRNA splicing [J]. Nucleic Acids Res.

[74] Jeck WR, Sharpless NE (2014). Detecting and characterizing circular RNAs [J]. Nat Biotechnol.

[75] Barrett SP, Salzman J. Circular RNAs: analysis, expression and potential functions [J]. Development (Cambridge, England). 2016,143(11):1838–47. 10.1242/dev.128074 PMC4920157 27246710

[76] Salzman J, Gawad C, Wang PL, Lacayo N, Brown PO (2012). Circular RNAs are the predominant transcript isoform from hundreds of human genes in diverse cell types [J]. PLoS One.

[77] AbouHaidar MG, Venkataraman S, Golshani A, Liu B, Ahmad T (2014). Novel coding, translation, and gene expression of a replicating covalently closed circular RNA of 220 nt [J]. Proc Natl Acad Sci U S A.

[78] Nilsson RJ, Balaj L, Hulleman E, van Rijn S, Pegtel DM, Walraven M (2011). Blood platelets contain tumor-derived RNA biomarkers [J]. Blood.

[79] Wang H, Xiao Y, Wu L, Ma D (2018). Comprehensive circular RNA profiling reveals the regulatory role of the circRNA-000911/miR-449a pathway in breast carcinogenesis [J]. Int J Oncol.

[80] Visvader JE, Lindeman GJ (2012). Cancer stem cells: current status and evolving complexities [J]. Cell Stem Cell.

[81] Yan N, Xu H, Zhang J, Xu L, Zhang Y, Zhang L (2017). Circular RNA profile indicates circular RNA VRK1 is negatively related with breast cancer stem cells [J]. Oncotarget.

[82] Gao D, Zhang X, Liu B, Meng D, Fang K, Guo Z (2017). Screening circular RNA related to chemotherapeutic resistance in breast cancer [J]. Epigenomics..

[83] Miao Y, Zheng W, Li N, Su Z, Zhao L, Zhou H (2017). MicroRNA-130b targets PTEN to mediate drug resistance and proliferation of breast cancer cells via the PI3K/Akt signaling pathway [J]. Sci Rep.

[84] Wang T, Shigdar S, Shamaileh HA, Gantier MP, Yin W, Xiang D (2017). Challenges and opportunities for siRNA-based cancer treatment [J]. Cancer Lett.

[85] Frazier KS (2015). Antisense oligonucleotide therapies: the promise and the challenges from a toxicologic pathologist's perspective [J]. Toxicol Pathol.

[86] Zhang Y, Xue W, Li X, Zhang J, Chen S, Zhang JL (2016). The biogenesis of nascent circular RNAs [J]. Cell Rep.

[87] Puttaraju M, Been MD (1992). Group I permuted intron-exon (PIE) sequences self-splice to produce circular exons [J]. Nucleic Acids Res.

[88] Li J, Yang J, Zhou P, Le Y, Zhou C, Wang S (2015). Circular RNAs in cancer: novel insights into origins, properties, functions and implications [J]. Am J Cancer Res.

[89] Lasda E, Parker R (2016). Circular RNAs co-precipitate with extracellular vesicles: a possible mechanism for circRNA clearance [J]. PLoS One.

[90] Hansen TB, Veno MT, Damgaard CK, Kjems J (2016). Comparison of circular RNA prediction tools [J]. Nucleic Acids Res.

[91] Patop IL, Kadener S (2018). circRNAs in Cancer [J]. Curr Opin Genet Dev.

[92] Zirkel A, Papantonis A. Detecting Circular RNAs by RNA Fluorescence In Situ Hybridization [J]. Methods in molecular biology (Clifton, NJ). 2018,1724:69–75. 10.1007/978-1-4939-7562-4_6 29322441

[93] Li Y, Chen B, Huang S. Identification of circRNAs for miRNA Targets by Argonaute2 RNA Immunoprecipitation and Luciferase Screening Assays [J]. Methods in molecular biology (Clifton, NJ). 2018,1724:209–18. 10.1007/978-1-4939-7562-4_17 29322452

[94] Du WW, Zhang C, Yang W, Yong T, Awan FM, Yang BB (2017). Identifying and characterizing circRNA-protein interaction [J]. Theranostics.

[95] Ghosal S, Das S, Sen R, Basak P, Chakrabarti J (2013). Circ2Traits: a comprehensive database for circular RNA potentially associated with disease and traits [J]. Front Genet.

[96] Liu YC, Li JR, Sun CH, Andrews E, Chao RF, Lin FM (2016). CircNet: a database of circular RNAs derived from transcriptome sequencing data [J]. Nucleic Acids Res.

[97] Dudekula DB, Panda AC, Grammatikakis I, De S, Abdelmohsen K, Gorospe M (2016). CircInteractome: a web tool for exploring circular RNAs and their interacting proteins and microRNAs [J]. RNA Biol.

[98] Chen X, Han P, Zhou T, Guo X, Song X, Li Y. circRNADb: A comprehensive database for human circular RNAs with protein-coding annotations [J]. Scientific reports. 2016,6:34985. 10.1038/srep34985 PMC5057092 27725737

[99] Xia S, Feng J, Chen K, Ma Y, Gong J, Cai F (2018). CSCD: a database for cancer-specific circular RNAs [J]. Nucleic Acids Res.

[100] Xia S, Feng J, Lei L, Hu J, Xia L, Wang J (2017). Comprehensive characterization of tissue-specific circular RNAs in the human and mouse genomes [J]. Brief Bioinform.

